# Molecular Cloning, Characterization, and Anti-avian Pathogenic *Escherichia coli* Innate Immune Response of the Cherry Valley Duck CIITA Gene

**DOI:** 10.3389/fmicb.2017.01629

**Published:** 2017-08-23

**Authors:** Rong Li, Mengjiao Guo, Jing Lin, Tongjie Chai, Liangmeng Wei

**Affiliations:** ^1^Sino-German Cooperative Research Centre for Zoonosis of Animal Origin of Shandong Province, College of Veterinary Medicine, Shandong Agricultural University Tai’an, China; ^2^Collaborative Innovation Center for the Origin and Control of Emerging Infectious Diseases, Taishan Medical University Tai’an, China; ^3^Shandong Provincial Key Laboratory of Animal Biotechnology and Disease Control and Prevention, Shandong Agricultural University Tai’an, China; ^4^Shandong Provincial Engineering Technology Research Center of Animal Disease Control and Prevention, Shandong Agricultural University Tai’an, China

**Keywords:** Cherry Valley duck, CIITA, sequence analysis, receptor expression, inflammatory cytokines, antibacterial ability, innate immunity

## Abstract

Class II major histocompatibility complex (MHC-II) transactivator (CIITA) is a member of the pattern recognition receptor in cytoplasm, which is involved in host innate immune responses. In this study, the full-length cDNA of Cherry Valley duck CIITA (duCIITA) was cloned from the spleen of healthy Cherry Valley ducks for the first time. The CDs of duCIITA have 3648 bp and encode 1215 amino acids. The homology analysis of CIITAs amino acid sequence showed that the duCIITA has the highest identity with the *Anas platyrhynchos* (94.9%), followed by *Gallus gallus* and *Meleagris gallopavo*. Quantitative real-time PCR analysis indicated that duCIITA mRNA has a broad expression level in healthy Cherry Valley duck tissues. It was highly expressed in the lung and cerebellum, and lowly expressed in the rectum and esophagus. After the avian pathogenic *Escherichia coli* (APEC) *O1K1* infection, the ducks exhibited the typical clinical symptoms, and a severe fibrinous exudate in the heart and liver surface was observed. Meanwhile, a significant up-regulation of duCIITA was detected in the infected liver. The inflammatory cytokines IL-1β, IL-6, and IL-8 have a significant up-regulation in the infected liver, spleen and brain. In addition, knockdown of the duCIITA reduces antibacterial activity and inflammatory cytokine production of the duck embryo fibroblast cells. Our research is the first study of the cloning, tissue distribution, and antibacterial immune responses of duCIITA, and these findings imply that duCIITA was an important receptor, which was involved in the early stage of the antibacterial innate immune response to APEC *O1K1* infection of Cherry Valley duck.

## Introduction

Janeway (1989) first proposed the concept of pattern recognition receptors (PRRs) in the immune system, expressed in phagocytic and other cells, which can recognize pathogen-associated molecular patterns (PAMPs), thus producing immune responses by stimulating the expression of downstream antibacterial or antiviral proteins. Nucleotide-binding oligomerization domain (NOD)-like receptors (NLRs) is an important class of intracellular PRRs, which recognize both exogenous and endogenous pathogens. It is generally accepted that NLRs play an important role in the regulation of the innate immune responses and resistance to pathogen invasion (Meylan et al., 2006). In mammals, most NLRs consist of three domains: the leucine-rich repeat (LRR) domain for ligand recognition, the nucleotide-binding and NACHT associated with the oligomerization and activation, and the N-terminal effector domain for signal transduction through protein–protein interactions (Koonin and Aravind, 2000; Ogura et al., 2001). Currently, the majority of studies have been constructed on NLRs and their functions in mammals. In waterfowl, such as ducks and geese, the most studied PRRs are the Toll-like receptors (TLRs) and retinoic acid-inducible gene I-like receptors (RLRs), with only a few studies on NLRs. However, the functions of PRRs in ducks are different from those in mammals to some extent. For example, in mammals, there is a TLR8 that can recognize single-stranded RNA viruses, whereas the ducks do not have this protein (MacDonald et al., 2008). There is a RIG-I receptor in ducks that can recognize the influenza virus, whereas chickens do not have this protein (Barber et al., 2010). The difference in their PRR profiles may lead to different sensitivity to the highly pathogenic avian influenza virus in chickens and ducks. Therefore, it is necessary to identify and clone the PRRs in waterfowl, which will lay a foundation for further understanding of the immune system.

In mammals, NLRs are able to recognize bacterial flagella, lipopolysaccharide, RNA, and muramyl dipeptides in the cytoplasm (Franchi et al., 2009). NLRs can be divided into five subfamilies according to the difference in the N-terminal effector domain (Ting and Davis, 2005). Class II major histocompatibility complex (MHC-II) transactivator (CIITA), first discovered in 1993, and contains an acidic transcriptional activation motif in the N-terminal domain, so it belongs to the NLRA subfamily. CIITA plays an important role in the MHC-II transcriptional activation and is positively correlated with the MHC-II transcription level (Muhlethaler-Mottet et al., 1997; Zuo and Rowe, 2012). In addition, CIITA can regulate the presentation function of antigen presentation cells by controlling the transcription level of MHC-II (van den Elsen et al., 2004; Lupfer and Kanneganti, 2012). However, CIITA does not directly bind to DNA but rather acts as a transcriptional co-activator through the activation of transcription factors (Sisk et al., 2003). In addition, CIITA can trans-activate the expression of MHC-II in antigen presentation cells and virus-infected target cells, inducing host-derived antiviral responses, thereby inhibiting the viral replication in the host and eliminating virus infection (Tosi et al., 2011).

Today, the bacterial pathogens pose a great threat to the duck industry worldwide. The incidence and mortality of ducks caused by various infectious bacterial pathogens such as *Salmonella enterica, Riemerella anatipestifer, Pasteurella multocida*, and *Escherichia coli* (*E. coli*) are higher than those caused by viruses (Wei B. et al., 2013). Avian pathogenic *E. coli* (APEC) are a pathogen of *E. coli* disease, which can infect ducks at different ages, mainly causing severe pericarditis, perihepatitis, and airsacculitis (Dho-Moulin and Fairbrother, 1999; Guabiraba and Schouler, 2015). At present, although there are many reports on the isolation and detection of this bacterium, the innate immune response to the host caused by the APEC infection remains unclear. Therefore, in this study, we cloned the CDs of the CIITA gene from Cherry Valley ducks (duCIITA) and examined its distribution in different tissues. We also investigated the mRNA expression level of the duCIITA and interleukins in the liver, spleen, and brain after the APEC *O1K1* infection. In addition, we knocked down the duCIITA in the duck embryo fibroblast cells (DEFs) to explore the antibacterial ability and role of duCIITA in the innate immunity. Our study will provide a foundation for the understanding of the innate immunity mechanism.

## Materials and Methods

### Cloning of the DuCIITA

In order to obtain the CDs of duCIITA, one set of specific polymerase chain reaction (PCR) primers was designed to identify the duCIITA sequences based on the predicated genes of chicken CIITA in the Genbank (**Table 1**). Total RNA (1 μg) was extracted from the spleen of healthy Cherry Valley ducks with the TRIzol reagent (Takara, Dalian, China) and the cDNA was obtained with the HiScript R II One-Step RT-PCR kit (Vazyme, Nanjing, China). The PCR conditions were pre-denatured 1 cycle of 94°C for 5 min; 35 cycles of 94°C for 30 s, 57°C for 30 s and 72°C for 4 min; and the final extension at 72°C for 10 min. Eventually, the full-length cDNA of duCIITA was sequenced by the Shanghai Invitrogen Biotechnology Co., Ltd.

**Table 1:** The primers information.

Primer name	Sequence of Oligonucleotide (5′–3′)	Purpose
F1	ATGAATCTTTTTAAGGAGAT	Gene cloning
R1	TTACACACTGATCCTCGAGT	Gene cloning
qβ-actin F	GGTATCGGCAGCAGTCTTA	qRT-PCR
qβ-actin R	TTCACAGAGGCGAGTAACTT	qRT-PCR
qCIITA F	CTGTTGCAGCATGTGACCTT	qRT-PCR
qCIITA R	TCTTCAGGCCAACCAAGTCT	qRT-PCR
qIL-1β F	TCATCTTCTACCGCCTGGAC	qRT-PCR
qIL-1β R	GTAGGTGGCGATGTTGACCT	qRT-PCR
qIL-6 F	TTCGACGAGGAGAAATGCTT	qRT-PCR
qIL-6 R	CCTTATCGTCGTTGCCAGAT	qRT-PCR
qIL-8 F	AAGTTCATCCACCCTAAATC	qRT-PCR
qIL-8 R	GCATCAGAATTGAGCTGAGC	qRT-PCR
qIL-10 F	GCCTCCACTTGTCTGACCTC	qRT-PCR
qIL-10 R	CCTCCATGTAGAACCGCATC	qRT-PCR

*qRT-PCR, quantitative real-time polymerase chain reaction; F, forward primer; R, reverse primer.*

### Sequence and Phylogenetic Analysis

The homology analysis of the duCIITA cDNA sequence was performed using the BLAST program of NCBI. The amino acid sequence of each species’ CIITAs was found on NCBI and the protein number is shown in **Table 2**. Meg Align software was used to analyze the similarity and evolution of the CIITAs’ amino acid sequence. The structure of the amino acid sequences of duCIITA was predicted by the SMART online tool^1^. Multiple amino acid sequence alignments were performed using ClustalW2^2^ and edited with the online tool Boxshade^3^. The phylogenetic analysis was generated using the MEGA5.0 software and the tree was constructed by the neighbor-joining method with the bootstrapping over 1000 replicates.

**Table 2:** Reference sequences information.

Species	GenBank accession numbers
*Canis lupus familiaris*	XP_005621608.1
*Leptonychotes weddelli*	XP_006734588.1
*Mustela putorius furo*	XP_012910604.1
*Sus scrofa*	AAM15722.1
*Orcinus orca*	XP_012388707.1
*Tursiops truncatus*	XP_019784137.1
*Equus caballus*	XP_014585603.1
*Loxodonta africana*	XP_010595614.1
*Cavia porcellus*	XP_012997031.1
*Ochotona princeps*	XP_004586810.1
*Saimiri boliviensis*	XP_010337670.1
*Homo*	NP_001273331.1
*Macaca fascicularis*	XP_005591301.1
*Papio anubis*	XP_009194239.1
*Mus musculus*	XP_006521787.1
*Rattus norvegicus*	NP_445981.2
*Alligator sinensis*	XP_014375177.1
*Pelodiscus sinensis*	XP_014427762.1
*Geospiza fortis*	XP_005420564.2
*Taeniopygia guttata*	XP_012432441.1
*Ficedula albicollis*	XP_005054348.1
*Columba livia*	XP_005508567.2
*Gallus*	XP_004945373.1
*Meleagris gallopavo*	XP_010717721.1
*Falco cherrug*	XP_005441588.2
*Anas platyrhynchos*	XP_012962329.1
*Oreochromis niloticus*	XP_005459511.1
*Salmo salar*	XP_014038785.1
*Clupea harengus*	XP_012682573.1
*Danio rerio*	XP_009297712.1
*Ictalurus punctatus*	AFL70283.1

### Bacteria, Cells and Animals

The bacterial strain of APEC *O1:K1* used in this study was stored by the Environmental Microbiology Laboratory in Shandong Agricultural University and was isolated from the clinical infected ducks that suffered from the colibacillosis. The bacterial strain was cultivated in Luria-Bertani (LB) agar at 37°C for 18 h. A single colony was inoculated in 5 mL of LB broth and cultivated at 37°C for 18 h with agitation.

Duck embryo fibroblast cells were derived from the 12-day-old Cherry Valley duck embryo and cultured in Dulbecco’s modified Eagle medium (DMEM) (Gibco, Grand Island, NY, United States), which included 10% fetal bovine serum (Transgen, Beijing, China), and was incubated at 37°C in an atmosphere with 5% (v/v) CO_2_.

One-day-old healthy Cherry Valley ducks were purchased from a farm in Tai’an, and housed in isolation for the experiment until 4 weeks old. The ducks were tested for several common diseases, such as Newcastle disease, avian influenza, duck Tembusu virus, and the duck plague virus, and were declared to be healthy. In addition, all the ducks used in this study were confirmed to be negative for the APEC *O1K1*.

### Animal Experiments

All ducks used in this experiment were handled according to the guidelines of the Ethics Committee on Animal Experiments of Shandong Agricultural University and the appropriate biosecurity guidelines, the number of the approval protocol being “SDAUA-2015-004.” At 4 weeks old, 3 healthy ducks were randomly selected and their tissues, including the heart, liver, spleen, lung, kidney, brain, duodenum, jejunum, ileum, cecum, rectum, cerebellum, thymus, bursa of Fabricius, muscular stomach, glandular stomach, trachea, esophagus, skin, and muscle were collected for the duCIITA tissue distribution analyses by quantitative real-time PCR (qRT-PCR).

To confirm whether the duCIITA was involved in the innate immune response caused by the APEC *O1K1*, 40 four-week-old Cherry Valley ducks were randomly divided into two groups. The experimental group was infected neck subcutaneously with 0.3 mL APEC *O1K1* (10^9^ CFU/mL) per duck, and the control group was inoculated with the same volume of 0.65% normal saline (Dozois et al., 2003). At 1, 2, and 3 days post-infection (dpi), three ducks with the significant clinical symptoms were killed, and the same numbers of ducks were also killed in the control group. A part of samples from the liver, spleen, and brain were treated with 10-fold serial dilution, then the diluent were plated onto eosin methylene blue nutrient agar and incubated at 37°C for 12 h to calculate intracellular bacterial CFU; a second part were fixed with 4% formalin solution at room temperature for histopathological examination; and another part of the samples were immediately preserved in liquid nitrogen for duCIITA and inflammatory cytokines detection by qRT-PCR. The clinical symptoms of the remaining ducks in the infected group were observed to 14 dpi before they were euthanized.

### SiRNA Interference

Three Si-CIITAs (pSi-CIITA-1, pSi-CIITA-2 and pSi-CIITA-3) sequences targeting the 2095–2116, 2217–2238, and 1371–1392 bp positions of duCIITA and negative control Si-RNA (pSi-NC) were synthesized by GenePharma (GenePharma, Shanghai, China). 1 μg pSi-CIITA or pSi-NC was transfected with *Trans*IL-LT1 Transfection Reagent to DEFs (Mirus Bio, CA, United States). After 24 h post-transfected (hpt), cell samples were directly collected for RNA extraction. PSi-NC acted as the control and the silencing efficiency of the Si-CIITAs was analyzed by qRT-PCR. The Si-RNA sequences are seen in **Table 3**.

**Table 3:** The sequences of pSi-RNA.

pSiRNA	Sense sequence (5′–3′)	Antisense sequence (5′–3′)
pSi-NC	UUCUCCGAACGUGUCACGUTT	ACGUGACACGUUAGAATT
pSi-CIITA-1	GGAGACAAAGGCCUUCCUUTT	AAGGAAGGCCUUUGUCUCCTT
pSi-CIITA-2	CCGUAUGCCUGGUAUCUUTT	AAGAUACCAGGCUAUACGGTT
pSi-CIITA-3	GCUGGUAACUUACAAUCUATT	UAGAUUGUAAGUUACCAGCTT

### Antibacterial Activity of DuCIITA

To investigate the antibacterial ability of duCIITA, DEFs were incubated in 6-well plates at 37°C. Before pSi-CIITA and pSi-NC transfection, the cells were growing with 80% confluent. After 24 hpt, DEFs were infected with 1 × 10^6^ CFU/mL APEC *O1K1* for 3 h, and then washed three times with PBS. The cells were then cultured with DMEM containing gentamicin (100 μg/mL) for 3 h to kill the extracellular APEC *O1K1*. The cell samples were collected with 500 μL 1% (v/v) Triton PBS and treated with 10-fold serial dilution. Then the processing of diluent was as above.

### Quantitative Real-Time PCR (QRT-PCR)

Total RNA of the above tissues and DEFs was extracted and reversed with the above method. QRT-PCR primers of duCIITA were designed based on the sequences of duCIITA obtained in this study with the primer3 software^4^ and selected by the dissociation curves. The qRT-PCR primers sequences of inflammatory cytokines IL-1β, IL-6, IL-8, and IL-10 are seen in **Table 1**. The duCIITA and ILs expression were normalized by the endogenous gene of β-actin (**Table 1**). QRT-PCR was performed using the ChamQ^TM^ SYBR^®^ qPCR Master Mix (Vazyme, Nanjing, China) and carried out with the 7500 Fast Real-Time PCR System (Applied Bio-systems, Foster City, CA, United States). The PCR consisted of 20 μL volume, with conditions as follows: one cycle of pre-denatured at 95°C for 5 min, followed by 40 cycles of denaturation at 95°C for 10 s and extension at 60°C for 34 s. Then, a dissociation curve was analyzed. Each sample was analyzed in triplicate.

### Calculations and Statistical Analysis

The relative expression levels of the duCIITA were detected using the duck β-actin gene as the endogenous reference gene and were calculated using the 2^-ΔΔC_T_^ method (Livak and Schmittgen, 2001). All data were represented as means ± standard deviations, and statistical analyses were performed by the Graph Pad Prism 5 software (Graph Pad Software Inc., San Diego, CA, United States). The differences were evaluated using Student’s *t*-test. A value of *P* < 0.05 was considered as significant and *P* < 0.01 was considered to be highly significant.

## Results

### Molecular Cloning, Structure, and Phylogenetic Analysis of DuCIITA

**FIGURE 1:**
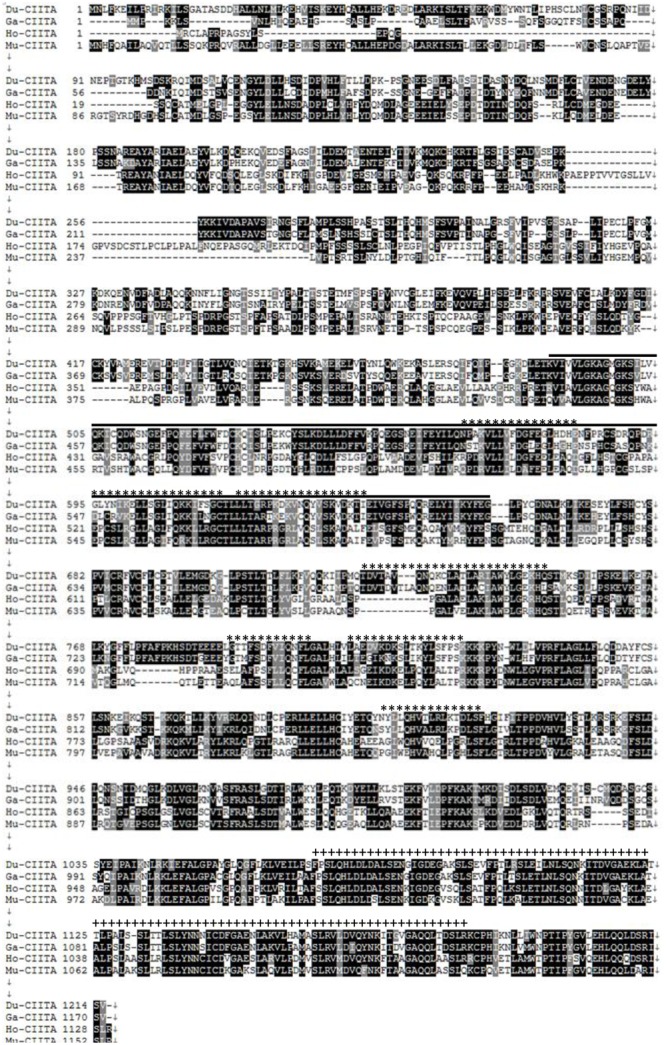
Multiple alignment of the Cherry Valley ducks CIITA. Alignment was performed using the Clustal X program and edited with Boxshade. The CIITA sequences are shown for Cherry Valley duck (Du), chicken (Ch), human (Hu), and mouse (Mu). The amino acid sequences used are seen in **Table 2**. Black shading indicates amino acid identity; gray shading indicates similarity (50% threshold); the black line represents the NACHT domain; the ^∗∗∗^ above the sequence represents the seven predicted transactivator domains; the +++ above the sequence represents the four LRR domains.

**FIGURE 2:**
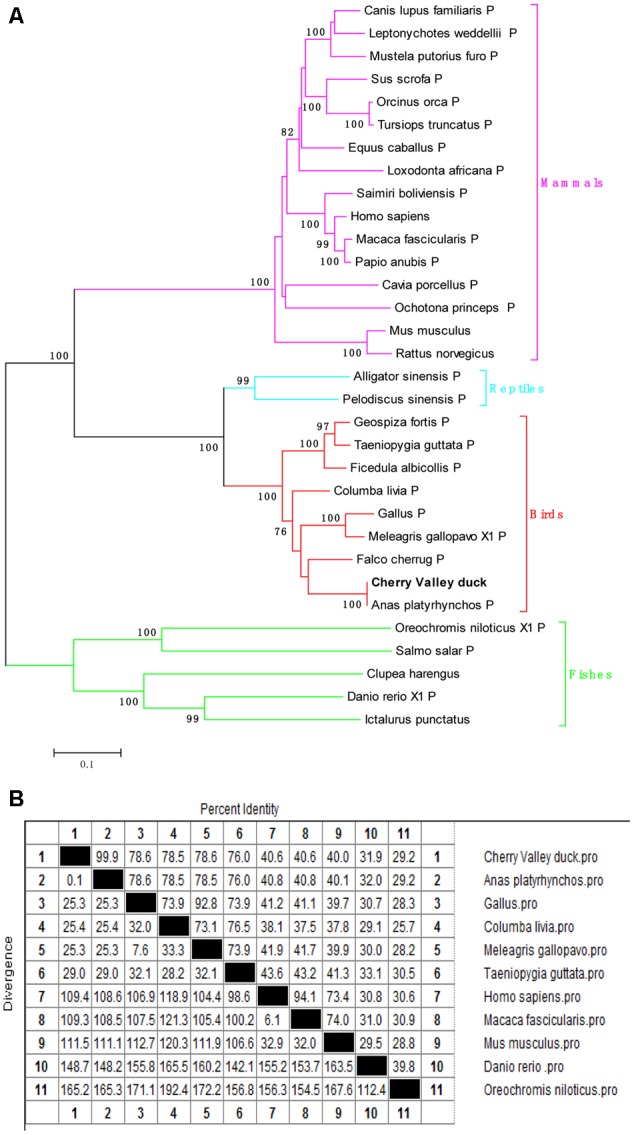
**(A)** A phylogenic tree based on CIITA between Cherry Valley duck and other species amino acid sequence. Neighbor-joining tree was generated using MEGA 5.0, and a 1000 bootstrap analysis was performed. The scale bar was 0.1. GenBank accession numbers were shown in **Table 2**. **(B)** Homology analysis of Cherry Valley duck CIITA in amino acid sequence (% amino acid sequence identities). Percent amino acid sequence homology was calculated by MegAlign software with Clustal W method. GenBank accession numbers were shown in **Table 2**.

The CDs of duCIITA consists of a 3648 bp and encodes 1215 amino acids. The duCIITA cDNA sequence showed a highly similar to the *Gallus gallus* CIITA. And the duCIITA contained characteristic domains of NLRs: a central NACHT domain and an LRR domain at its C-terminus (**Figure 1**). Multiple alignment analysis showed that duCIITA had seven MHC II transactivator domains in 564–582, 594–615, 618–638, 724–750, 790–802, 809–826, and 902–917 residues (**Figure 1**). The phylogenetic tree indicated that there were four major branches: mammals, reptiles, birds, and fishes (**Figure 2A**). The duCIITA and *Falco cherrug* CIITA were in the same small branch, which has the closest relationship. However, the genetic relationship between duCIITA has the most distant relationship with fishes (**Figure 2A**). DuCIITA had the highest identity to the *Anas platyrhynchos* (94.9%), 78.6% identity with *G. gallus* and *Meleagris gallopavo* (**Figure 2B**). But the duCIITA amino acid sequence showed a relatively lower identity with mammals CIITAs, only 29.5–40.6% (**Figure 2B**). These results showed that the duCIITA amino acid sequence was closely related to the CIITAs of other birds.

### Clinical Symptoms, Necropsy Lesions and Histopathological Analysis in the Infected Tissues

**FIGURE 3:**
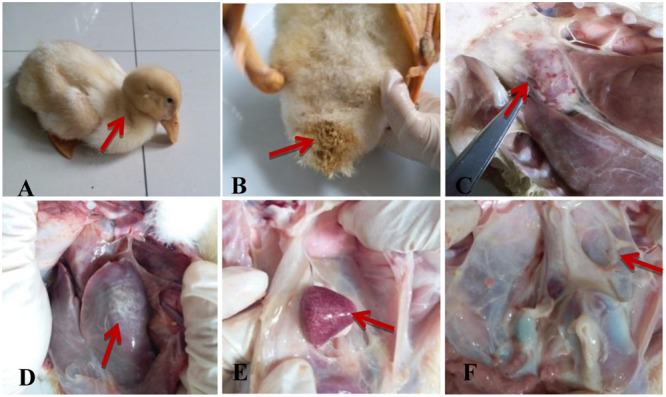
Clinical symptoms and necropsy lesions of the APEC-infected ducks. **(A)** Listlessness and anorexia; **(B)** Diarrhea; **(C)** Heart surface had a layer of yellowish–white fibrinous exudate; **(D)** Fibrinous exudate in the liver surface; **(E)** Splenomegaly and hemorrhage; **(F)** Peritoneal fibrosis adhesions.

The ducks infected with APEC *O1K1* exhibited the typical clinical symptoms, including listlessness, anorexia (**Figure 3A**), and diarrhea (**Figure 3B**). At necropsy, the heart and liver surface had a layer of yellowish–white fibrinous exudate attachment (**Figures 3C,D**), splenomegaly and hemorrhage (**Figure 3E**), and peritoneal fibrosis adhesions (**Figure 3F**). One duck died at 4 dpi in the infected group, and the condition of the remaining ducks was improved. Finally, they were euthanized at 14 dpi. No clinical symptoms or necropsy lesions were observed in the control group throughout the experiment. In addition, the *E. coli* rapid replication in the liver, spleen, and brain at 1 dpi, and reached its peak at 2 dpi with 10^8^ CFU/g. However, the bacteria content of all the tested tissues dramatically declined at 3 dpi in our previous study (Li et al., 2016).

**FIGURE 4:**
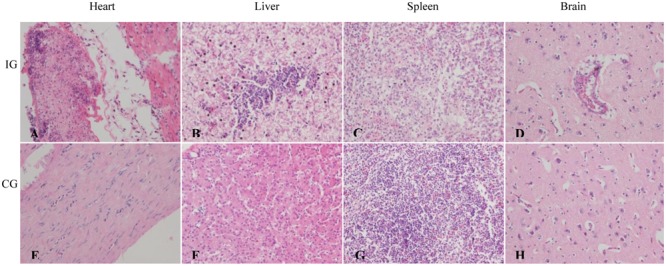
Pathological changes of the APEC-infected ducks. **(A)** Heart: a large amount of fibrinous exudate, necrosis heterophilic granulocyte and histiocytic cells in the caseous exudate. **(B)** Liver: diffuse vacuolar degeneration and a lot of necrosis of hepatocyte with lymphocytic infiltration. **(C)** Spleen: tissue loose, lymphocytic necrosis and heterophilic granulocyte infiltration. **(D)** Brain: perivascular inflammatory infiltrates. **(A–D)** Represent the heart, liver, spleen, and brain of ducks from the APEC-infected group, respectively; **(E–H)** represent the heart, liver, spleen, and brain of ducks from the control group, respectively. Magnification 400×. IG, infection group; CG, control group.

As shown in **Figure 4A**, there was an obvious fibrinous exudate in the epicardial surface and a large amount of necrotic heterophilic granulocyte and histiocytic cells were revealed in the caseous exudate. In the liver, the infected ducks displayed a severe diffuse vacuolated degeneration, a serious hepatocellular necrosis, and lymphocyte infiltration (**Figure 4B**). In the spleen, the tissue were loose, lymphocytic necrosis, and heterophilic granulocyte infiltration was discovered (**Figure 4C**). No obvious microscopic lesions were observed in the brains of the infected ducks, except for the perivascular inflammatory infiltrates (**Figure 4D**). In addition, no obvious microscopic lesions were discovered in the corresponding tissues from the control group (**Figures 4E–H**).

### Tissue Distribution of DuCIITA

**FIGURE 5:**
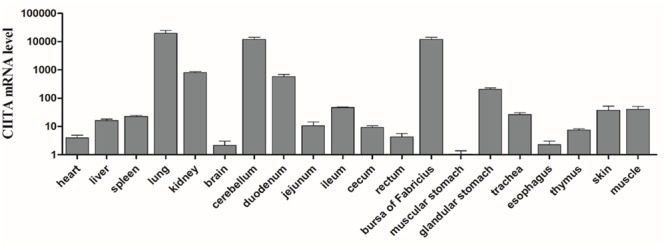
Tissue distributions of CIITA in the healthy Cherry Valley duck. The relative mRNA levels were normalized to the expression of the β-actin gene from various tissues. The data were normalized to the muscular stomach, and error bars indicated the SD.

To investigate the expression profile of duCIITA mRNA in normal tissue, 20 kinds of tissues were separated from 3 four-week-old healthy ducks. The muscular stomach was chosen as the standard tissue, and the duCIITA mRNA was expressed in all the tested tissues (**Figure 5**). It was strongly expressed in the lung and cerebellum, and weakly expressed in the rectum and esophagus (**Figure 5**). The wide expression of duCIITA indicates that the duCIITA might be extensively involved in the host immune response of healthy Cherry Valley ducks.

### Expression Profiles of DuCIITA in the Infected Ducks

**FIGURE 6:**
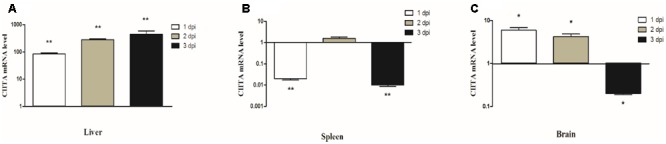
Relative mRNA expression levels of duCIITA in liver, spleen, and brain of the APEC-infected ducks. **(A)** Liver, **(B)** Spleen, **(C)** Brain. The fold change was calculated by the experimental ducks vs. control ducks at the same time point, using the 2^-ΔΔC_T_^ method. All data were expressed as means ± SD (*n* = 3), and Student’s *t*-test was performed to evaluate the differences. ^∗^Significant difference (*P* < 0.05); ^∗∗^highly significant difference (*P* < 0.01); dpi, days post-infection.

To confirm whether duCIITA was involved in the host antibacterial responses, we investigated the mRNA expression of duCIITA in the liver, spleen, and brain during APEC *O1K1* infection. In the liver, the mRNA expression of duCIITA was highly significantly increased in a short time, and increased to 83.4- and 277.9-fold at 1 and 2 dpi, respectively (*P* < 0.01). Then, it reached a peak at 3 dpi (445.5-fold, *P* < 0.01) (**Figure 6A**). In the spleen, the expression level of duCIITA was significantly down-regulated at 1 and 3 dpi (*P* < 0.01) and increased a little at 2 dpi (1.6-fold, *P* > 0.05) (**Figure 6B**). In the brain, the expression of duCIITA was significantly up-regulated at 1 and 2 dpi (5.9- and 4.2-fold, respectively, *P* < 0.05), and down-regulated at 3 dpi (*P* < 0.05) (**Figure 6C**). These results suggest that duCIITA may be involved in the host innate immunity to APEC *O1K1* infection.

### Expression Profiles of Inflammatory Cytokines in the Bacteria (APEC *O1K1*) of Infected Ducks

**FIGURE 7:**
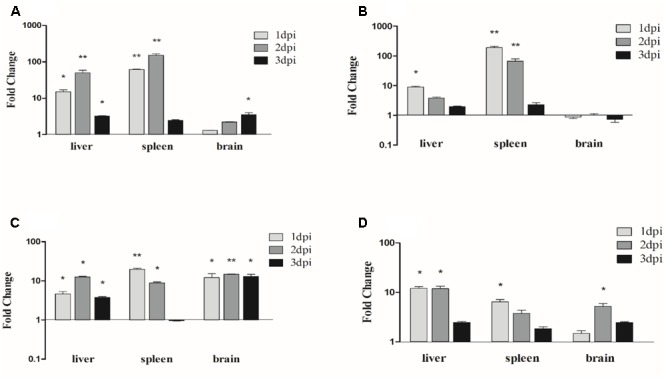
Expression profiles of inflammatory cytokines in the APEC-infected ducks. **(A)** The expression profiles of IL-1β in liver, spleen, and brain post-infection with APEC, **(B)** the expression profiles of IL-6, **(C)** the expression profiles of IL-8 and **(D)** the expression profiles of IL-10. The samples of APEC-infected and control ducks were collected at 1, 2, and 3 dpi. And the fold change was calculated by the experimental ducks vs. control ducks at the same time point, using the 2^-ΔΔC_T_^ method. All data were expressed as means ± SD (*n* = 3), and Student’s *t*-test was performed to evaluate the differences. ^∗^Significant difference (*P* < 0.05); ^∗∗^highly significant difference (*P* < 0.01); dpi, days post-infection.

To identify the inflammatory cytokines could involve in the antibacterial innate immune response of the APEC *O1K1* infected ducks, the mRNA expressions of IL-1β, IL-6, IL-8, and IL-10 in the liver, spleen, and brain were measured by the qRT-PCR method. As shown in **Figure 7A**, the proinflammatory cytokines IL-1β mRNAs expression levels were all up-regulated in the liver, spleen, and brain during the three tested days. Among them, the highest up-regulated level was in the spleen with 151.1-fold at 2 dpi (*P* < 0.01) (**Figure 7A**), and the expression of IL-1β also peaked with 50.4-fold at 2 dpi in the liver (*P* < 0.01) (**Figure 7A**). In the brain, the mRNA expression of IL-1β had a sustained up-regulation at 1–3 dpi (**Figure 7A**). The proinflammatory cytokine IL-6 mRNA expression level also showed up-regulated at most of the time in the tested tissues. The greatest increase of IL-6 was in the spleen at 2 dpi (191.1-fold, *P* < 0.01) (**Figure 7B**); in the liver, similar to the spleen, the mRNA expression trend of IL-6 reached its peak at 1 dpi with 8.9-fold (*P* < 0.05) (**Figure 7B**), and then the up-regulated times gradually decreased (**Figure 7B**); however, the mRNA expression of IL-6 in the brain always remained at a background level at 1–3 dpi (**Figure 7B**). Unlike IL-6, the mRNA expression of proinflammatory cytokine IL-8 had a stable and significant up-regulation in the brain (**Figure 7C**), and in the spleen, was found to be significantly up-regulated at 1 dpi with 19.6-fold (*P* < 0.01) (**Figure 7C**). As one of the major suppressible inflammatory cytokines, the IL-10 mRNA expression also had a different degree of increase in the liver, spleen, and brain; among them, the highest up-regulation was expressed in the liver with 12.1-fold at 1 dpi (*P* < 0.05) (**Figure 7D**).

### DuCIITA Knockdown Reduces the Antibacterial Activity and Inflammatory Cytokine Production of DEFs

**FIGURE 8:**
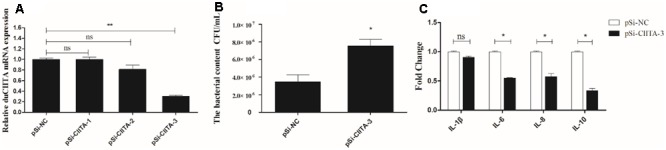
DuCIITA knockdown decreases antibacterial activity and inflammatory cytokine production in DEFs. **(A)** The DEFs were seeded in 6-well plates and transfected with 1.0 μg/well of pSi-RNA. After 24 hpt, the cells were collected for duCIITA detection. **(B)** The DEFs were infected with 1 × 10^6^ CFU/mL APEC *O1K1* at 24 hpt, the cells were lysed with 10-fold serial dilution and plated onto eosin methylene blue nutrient agar to calculate intracellular bacterial CFU. **(C)** The DEFs were infected with 1 × 10^6^ CFU/mL APEC *O1K1* at 24 hpt, then cells were collected for inflammatory cytokine detection. All data were expressed as means ± SD (*n* = 3), and Student’s *t*-test was performed to evaluate the differences. ^∗^Significant difference (*P* < 0.05); ^∗∗^highly significant difference (*P* < 0.01).

To further explore the antibacterial function of duCIITA, DEFs were transfected with pSi-CIITA or pSi-NC for 24 h. The cells were then collected for silencing efficiency analysis. As shown in **Figure 8A**, both pSi-CIITA-2 and pSi-CIITA-3 were able to decrease the mRNA expression of duCIITA in DEFs, but the pSi-CIITA-3 had stronger interference ability (**Figure 8A**), so we chose the pSi-CIITA-3 for further study. DEFs were infected with APEC *O1K1* after transfection, and then processed as above. The number of *E. coli* in DEFs transfected with pSi-CIITA was significantly higher than with the pSi-NC group (*P* < 0.05) (**Figure 8B**). In addition, after 24 hpt, cells were infected with 1 × 10^6^ CFU/mL *E. coli* and collected for inflammatory cytokine detection. As shown in **Figure 8C**, the expression of inflammatory cytokines IL-6, IL-8, and IL-10 in pSi-CIITA group was significantly lower than that in the pSi-NC group; however, there was no significant change in the IL-1β mRNA expression. These results suggest that the knockdown of duCIITA could weaken the antibacterial activity and reduce the inflammatory cytokine production of DEFs.

## Discussion

The plant disease resistance genes (R genes) can recognize and prevent the spread of pathogens such as fungi, viruses, and parasites (Dangl and Jones, 2001). NLRs may be the most ancient PRR family with similar structures and functions to the R genes (DeYoung and Innes, 2006). When the C-terminal LRR motif of NLRs recognizes the corresponding PAMPs, the PAMPs can bind directly or indirectly to the LRR motifs, thereby causing the conformational change of the NLRs, exposing the NACHT domain, triggering oligomerization, and exposing the N-terminal functional domain (Lange et al., 2011). The effector domain recruits adaptors and signaling proteins through homotypic interaction and then activates the downstream signaling pathways. At present, at least 23 and 34 NLRs have been discovered in humans and mice, respectively (Chen et al., 2009).

In this study, the full-length CDs of duCIITA were obtained. Predicted by the SMART tool, we found that the duCIITA contained typical NACHT and LRR motifs of the NLRs (**Figure 1**). Evolutionary analysis showed that the duCIITA has a closely genetic relationship to the CIITA of *A. platyrhynchos* (**Figure 2A**). However, the duCIITA was remotely related to mammals, and the similarity with the fishes was the least (**Figure 2B**). The duCIITA is widely distributed in healthy duck tissues, but the expression level in various tissues differs. Our results show that duCIITA has the highest expression level in the lung and the lowest in the stomach (**Figure 5**). Brujeni and Khosravi (2015) successful cloned CIITA from broilers and found it widely expressed in the liver, spleen, kidney, and thymus, and the expression of the CIITA mRNA significantly increased in the spleen after the *Brucella* infection. Thus, we had chosen the liver, spleen, and brain as the representatives of the target, immune, and nerve organ, respectively, for duCIITA detection after APEC *O1K1* infection. The liver was the main target organ of APEC *O1K1* infection, where the expression of duCIITA was kept elevated and reached the peak at 3 dpi (**Figure 6A**). This is consistent with previous studies, which showed that the expression of CIITA is up-regulated in chicken, mice, and catfish after the bacteria infection (Shtrichman et al., 2002; Liu et al., 2012; Brujeni and Khosravi, 2015). The innate immune response induced by the same pathogenic microbes was not consistent in the different cells, so we suspected this is one of the reasons for the different duCIITA expression in the liver, spleen, and brain. In addition, the unique function and susceptibility of these organs to the APEC infection maybe another reasons for the different duCIITA expression. Although the true reasons for the different duCIITA expression in the tested tissues were also unclear, but these results suggested that duCIITA could participate in the antibacterial immune process of *E. coli* infection.

In the current study, the APEC *O1K1* could rapidly replicate at the early stage of infection both in liver, spleen, and brain. However, the duCIITA expression displayed a significant difference in these tissues. So, we suspected there is not a simply positive correlation with the duCIITA expression and bacterial colonization. Firstly, the diverse cells in these organs may be due to the distinct innate immune response and the different duCIITA expression levels. Secondly, except the duCIITA, there also have a various other kinds of PRRs which can sense the pathogenic microbes in the host cells, so after the APEC *O1K1* infection, the diverse amounts and kinds of PRRs in the distinct tissues may result in the different expression level of duCIITA.

Studies have shown that NLRs are closely related to the pathogenesis of allergic diseases and autoimmune diseases as well as many other diseases (Rasmussen et al., 2001). When the microbial infection occurs, the NLRs could rapidly initiate the innate immunity of the organism. By sensing the PAMPs, they promote the downstream secretion of interferon and cytokines, and activate the expression of antibacterial or antiviral proteins (Meylan et al., 2006). In addition, NLRs can also assist the adaptive immune process, which has great significance to the removal of foreign pathogenic microorganisms (Janeway, 1989; Akira et al., 2006). In this study, the proinflammatory cytokines were significantly up-regulated in the tested tissues. Among them, the most significantly up-regulated was discovered in the spleen; for example, the IL-1β expression peaked with 151.1-fold at 2 dpi in the spleen (**Figure 7A**), and IL-6 and IL-8 expressions also reached their peaks at 1 dpi with 191.1- and 19.6-fold, respectively (**Figures 7B,C**). These results imply that the APEC *O1K1* infection has caused the inflammatory reaction in the host. Studies have pointed out that highly significant up-regulation of IL-6 and IL-8 could lead to more deaths in infected mammals and ducks (Chan et al., 2005; Wei L. et al., 2013). However, in our study, what is worth noting is the suppressible inflammatory cytokine IL-10 also has a significant up-regulated in the tested tissues (**Figure 7D**), and this might explain the phenomenon that the infection of remaining ducks were gradually alleviated at the later stages of the infection.

In order to determine whether the duCIITA had the antibacterial function, pSi-CIITA was constructed to knock down the duCIITA *in vitro*. Results showed that the bacterial content in DEFs transfected with pSi-CIITA was significant higher than those of DEFs transfected with pSi-NC (**Figure 8B**). This indicated that the duCIITA has an antibacterial function in the APEC *O1K1-*infected DEFs. To further confirm the role of duCIITA in inflammatory response, the inflammatory cytokines IL-1β, IL-6, IL-8, and IL-10 were detected in the transfected-infected DEFs. The expression of inflammatory cytokines IL-6, IL-8, and IL-10 in the pSi-CIITA group was significantly lower than that in the pSi-NC group (**Figure 8C**). Studies have shown that other members of NLRs, such as NOD1, NOD2, and NLRP3 could activate the CARD9, NF-κB, and caspase-1-mediated apoptotic pathway, respectively, for the cleavage and production of IL-1β and IL-18 (Lamkanfi et al., 2007; Kufer and Sansonetti, 2011; Sha et al., 2014). These results suggested that the knockdown of duCIITA could reduce the production of inflammatory cytokines and that the duCIITA plays an important regulatory role in the innate immune response caused by APEC *O1K1* infection.

To date, there is a growing body of research on PRRs in waterfowl, but the interaction between PRRs and PAMPs and its signal transduction pathways are still unclear. Based on the crucial role of the CIITA in the antibacterial immunity, we cloned and sequenced the duCIITA, predicted its main functional domains, and studied the antibacterial function of duCIITA in APEC *O1K1*-infected tissues and cells. This study will lay a foundation for the understanding of the mechanism of the antibacterial innate immunity.

## Author Contributions

LW contributed to the conception of the study. RL and MG performed most of the experiments, data analysis and wrote the manuscript. RL and JL contributed significantly to analysis and manuscript preparation. TC helped perform the analysis with constructive discussions.

## Conflict of Interest Statement

The authors declare that the research was conducted in the absence of any commercial or financial relationships that could be construed as a potential conflict of interest.

## Footnotes

1 http://smart.embl-heidelberg.de/

2 http://www.ebi.ac.uk/Tools/clustalw2/index.html

3 http://www.ch.embnet.org/software/BOX_form.html

4 http://bioinfo.ut.ee/primer3-0.4.0/
