# Corrigendum: Molecular Cloning, Characterization, and Anti-avian Pathogenic *Escherichia coli* Innate Immune Response of the Cherry Valley Duck CIITA Gene

**DOI:** 10.3389/fmicb.2017.02172

**Published:** 2017-10-31

**Authors:** Rong Li, Mengjiao Guo, Jing Lin, Tongjie Chai, Liangmeng Wei

**Affiliations:** ^1^Sino-German Cooperative Research Centre for Zoonosis of Animal Origin of Shandong Province, College of Veterinary Medicine, Shandong Agricultural University, Tai'an, China; ^2^Collaborative Innovation Center for the Origin and Control of Emerging Infectious Diseases, Taishan Medical University, Tai'an, China; ^3^Shandong Provincial Key Laboratory of Animal Biotechnology and Disease Control and Prevention, Shandong Agricultural University, Tai'an, China; ^4^Shandong Provincial Engineering Technology Research Center of Animal Disease Control and Prevention, Shandong Agricultural University, Tai'an, China

**Keywords:** Cherry Valley duck, CIITA, sequence analysis, receptor expression, inflammatory cytokines, antibacterial ability, innate immunity

In the original article Accolla et al. (1985, 1986) were not cited in the article. In addition, we erroneously stated the years in which CIITA was discovered; it should have been 1985–1986. The citation and correct date have been inserted in introduction, second paragraph.

The corrected paragraph should read:

In mammals, NLRs are able to recognize bacterial flagella, lipopolysaccharide, RNA, and muramyl dipeptides in the cytoplasm (Franchi et al., 2009). NLRs can be divided into five subfamilies according to the difference in the N-terminal effector domain (Ting and Davis, 2005). Class II major histocompatibility complex (MHC-II) transactivator (CIITA), discovered in 1985–1986 (Accolla et al., 1985, 1986), and contains an acidic transcriptional activation motif in the N-terminal domain, so it belongs to the NLRA subfamily. CIITA plays an important role in the MHC-II transcriptional activation and is positively correlated with the MHC-II transcription level (Muhlethaler-Mottet et al., 1997; Zuo and Rowe, 2012). In addition, CIITA can regulate the presentation function of antigen presentation cells by controlling the transcription level of MHC-II (van den Elsen et al., 2004; Lupfer and Kanneganti, 2012). However, CIITA does not directly bind to DNA but rather acts as a transcriptional co-activator through the activation of transcription factors (Sisk et al., 2003). In addition, CIITA can trans-activate the expression of MHC-II in antigen presentation cells and virus-infected target cells, inducing host-derived antiviral responses, thereby inhibiting the viral replication in the host and eliminating virus infection (Tosi et al., 2011).

The authors apologize for this error and state that this does not change the scientific conclusions of the article in any way.

## Conflict of interest statement

The authors declare that the research was conducted in the absence of any commercial or financial relationships that could be construed as a potential conflict of interest.
